# Long noncoding RNA DANCR confers cytarabine resistance in acute myeloid leukemia by activating autophagy *via* the miR‐874‐3P/ATG16L1 axis

**DOI:** 10.1002/1878-0261.12661

**Published:** 2021-02-27

**Authors:** Hao Zhang, Ling Liu, Lulu Chen, Haihui Liu, Saisai Ren, Yanling Tao

**Affiliations:** ^1^ Department of Hematology Affiliated Hospital of Jining Medical University China; ^2^ Graduate School Jining Medical University China; ^3^ Department of Pediatric Hematology Affiliated Hospital of Jining Medical University China

**Keywords:** acute myeloid leukemia, autophagy, cytarabine resistance, DANCR, long noncoding RNA, miR‐874‐3P/ATG16L1 axis

## Abstract

Autophagy is an important mechanism involved in the regulation of acute myeloid leukemia (AML) chemoresistance. The long noncoding RNA (lncRNA) differentiation antagonizing non‐protein coding RNA (DANCR) exhibits oncogenic activity in several types of human cancers, including AML, but it remains unclear whether it regulates autophagy and chemoresistance in AML. We report here that cytarabine (Ara‐C) treatment elevates DANCR expression in human AML cells. In addition, DANCR overexpression confers and its knockdown diminishes Ara‐C resistance in human AML cells, suggesting that DANCR positively regulates AML chemoresistance to Ara‐C. Moreover, DANCR promotes autophagy in Ara‐C‐treated human AML cells and acts as a sponge to decrease miR‐20a‐5p expression, thereby upregulating the expression of ATG16L1, a critical component of the autophagy machinery. Importantly, ATG16L1 silencing abrogates DANCR‐promoted autophagy and markedly restores DANCR‐conferred Ara‐C resistance, suggesting that DANCR promotes MIR‐874‐3P/ATG16L1 axis‐regulated autophagy to confer Ara‐C resistance in human AML cells. Together, this study identifies DANCR as a positive regulator of Ara‐C resistance in human AML cells, suggesting this lncRNA as a potential target for overcoming Ara‐C resistance in AML chemotherapy.

## Graphical abstract

Cytarabine (Ara‐C) induces the expression of differentiation antagonizing non‐protein coding RNA in acute myeloid leukemia cells, which acts as a ceRNA to directly bind and sponge miR‐874‐3P. This relieves inhibition of ATG16L1 mRNA and induces autophagy, which in turn mediates chemoresistance to Ara‐C.

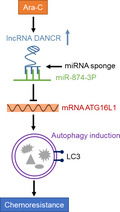

**Abbreviations**
AML: acute myeloid leukemia
Ara‐C: cytarabine
ceRNA: competing endogenous RNA
DANCR: differentiation antagonizing non‐protein coding RNA
lncRNA: long noncoding RNA
miRNA: microRNA
NC: negative control
WT: wild‐type

## 1 Introduction

Acute myeloid leukemia (AML) is an aggressive hematopoietic malignancy caused by the malignant transformation of hematopoietic stem and/or progenitor cells (Dohner *et al.*, 2015). Despite rapid progress in the development of novel therapeutic approaches, chemotherapy composed of cytarabine (Ara‐C) and an anthracycline remains the standard of care for AML over the past three decades (Roboz, 2011; Schenk *et al.*, 2012). Chemotherapy alone cures approximately 35–40% of younger AML patients, whereas for older patients > 60 years of age, the prognosis is even worse and the long‐lasting remission rate is only about 15% (Dohner *et al.*, 2010; Sill *et al.*, 2011). This inefficiency is mainly attributed to chemoresistance that leads to the development of refractory disease (Dohner *et al.*, 2015; Krug *et al.*, 2011). Therefore, chemoresistance represents one of the prominent targets in AML chemotherapy, and overcoming it holds a tremendous potential to increase survival of AML patients (Zebisch *et al.*, 2016).

In recent years, autophagy, a lysosomal self‐degradation process of aggregated proteins and damaged organelles (Kondo *et al.*, 2005), has been found to participate in several aspects of AML, including development (Liu *et al.*, 2016), differentiation (Orfali *et al.*, 2012), proliferation (Watson *et al.*, 2015), survival (Altman *et al.*, 2014), and particularly chemoresistance (Zhang *et al.*, 2013). For instance, some studies have shown that autophagy confers resistance to inhibitors of bromodomain and extraterminal domain and FLT‐3 tyrosine kinase in AML cells (Hanekamp *et al.*, 2014; Jang *et al.*, 2017), and conversely, inhibition of autophagy enhances AML sensitivity to chemotherapy of Ara‐C and cytosine arabinoside (Kim *et al.*, 2014; Nourkeyhani *et al.*, 2016). These findings point to an important role of autophagy involved in AML chemoresistance, showing promise for exploring autophagy as a therapeutic target in improving AML outcome.

The long noncoding RNAs (lncRNAs), a class of transcripts (> 200 nt) containing no or limited protein‐coding potential, are emerging as important players in cancer biology, demonstrating both oncogenic and tumor‐suppressive activities through multiple mechanisms, such as acting as a sponge of microRNAs (miRNAs) and regulating autophagy (Gibb *et al.*, 2011; Gutschner and Diederichs, 2012; Xu *et al.*, 2017). Lately, the lncRNA differentiation antagonizing non‐protein coding RNA (DANCR) was found to exhibit a wide spectrum of oncogenic activities in several types of cancers (Thin *et al.*, 2018). Further, it is also reported that in AML, DANCR has a functional role in the self‐renewal, quiescence, and engraftment of AML stem cells (Bill *et al.*, 2019). Regardless of this knowledge, whether DANCR regulates AML chemoresistance is still not yet investigated. In this study, we demonstrate that DANCR confers Ara‐C resistance in AML, in which the promoted activation of autophagy *via* MIR‐874‐3P/ATG16L1 axis plays a vital role.

## 2 Materials and methods

### 2.1 AML cells and culture

The human AML cell lines HL60, U937, and KG1a were obtained from American Type Culture Collection (Manassas, VA, USA). Clinical AML peripheral blood mononuclear cells were isolated from bone marrow of four *de novo*‐diagnosed pediatric AML patients as described previously (Bossis *et al.*, 2014). Isolation was performed using Ficoll density gradient centrifugation (GE Healthcare, Piscataway, NJ, USA) according to the manufacturer's instructions. The signed informed consent was obtained from each patient in accordance with the Declaration of Helsinki (World Medical Association, 2001). The study protocols were approved by the Ethics Committee of Affiliated Hospital of Jining Medical University. Throughout this study, all AML cells were cultured in complete RPMI 1640 medium supplemented with 10% FBS and 100 U·mL^−1^ penicillin/streptomycin (Thermo Fisher Scientific, Waltham, MA, USA) at 37 °C in an atmosphere of 5% CO_2_.

### 2.2 Cell treatment and transfection

One day before treatment, AML cells were seeded with a density of 2 × 10^5^ cells·mL^−1^. Fresh medium containing different concentrations of Ara‐C (Selleck, S1648, Houston, TX, USA) was added into the culture medium. Cells were further cultured for 24 or 48 h based on experimental purposes. For inhibition of autophagy, HL‐60 cells were treated with 200 nm bafilomycin A1 (Selleck, S1413) or 10 µm chloroquine (Selleck, S4157). Overexpression and knockdown were performed by transfection. HL60 and U937 cells were transfected with pcDNA3.1 vector, pcDNA3.1‐DANCR, miR‐NC, miR‐874‐3P mimic, nontarget antagomir, or miR‐874‐3P antagomir for overexpression using the FuGENE 6 Transfection Reagent (Promega, Madison, WI, USA), or transfected with siRNA control, siDANCR, siATG16L1, siSox4, or siSall4 for knockdown using the Lipofectamine RNAiMAX Reagent (Thermo Fisher Scientific), according to the manufacturer's protocol. At 48 or 72 h after transfection, cells were harvested for further biochemical analyses.

### 2.3 Cell viability and apoptosis detection

The viability of AML cells was determined by MTT assay using the CellTiter Non‐Radioactive Cell Proliferation Assay Kit (Promega) according to the manufacturer's protocol (Chang *et al.*, 2016). Briefly, after treatment, MTT (20 µL) was added into each well and cells were further incubated for 4 h at 37 °C. Then, DMSO (150 µL) was added into each well to dissolve formazan precipitate. The value of absorbance at 490 nm was read by the SpectraMax M5 Microplate Reader (Molecular Device, Sunnyvale, CA, USA). Cell apoptosis was determined by double staining with annexin V‐FITC and propidium iodide (PI), followed by cytometry analysis using a FACSCalibur flow cytometer (BD Biosciences, San Jose, CA, USA). Annexin V‐positive cells were defined as apoptotic cells, including those in early (annexin V+PI−) and late (annexin V+PI+) phases of apoptosis.

### 2.4 qRT‐PCR analysis

Total RNA was isolated using the TRIzol reagent (Thermo Fisher Scientific), and then, the complementary cDNA was synthesized using the RevertAid First Strand cDNA Synthesis Kit (Thermo Fisher Scientific) according to the manufacturer's instructions. qRT‐PCR analysis of DANCR and miRNAs was performed using the CFX96 PCR System (Bio‐Rad, Hercules, CA, USA). β‐Actin or U6 was used as a reference or nontarget control. The primers used in this study are available upon request.

### 2.5 Immunoblotting and immunofluorescence

Immunoblotting was performed as described previously (Sujobert *et al.*, 2015). Briefly, equal amount of proteins from each sample (30 µg) was separated by 10% or 12% SDS/PAGE, transferred to PVDF membranes, and then probed with primary and corresponding secondary antibodies. Primary antibodies against LC3, ATG5, and ATG16L1 were purchased from Novus Biologicals. Primary antibodies against Bax, ATG7, and β‐actin, and secondary antibodies were purchased from Abcam. Primary antibodies against cleaved caspase‐3, Bcl‐2, and Beclin‐1 were purchased from Cell Signaling. Protein band was developed using the ECL chemiluminescence reagent (GE Healthcare). The intensity of protein band was analyzed by imagej software (National Institutes of Health, Bethesda, MD, USA). The LC3 puncta in HL60 cells was detected by immunofluorescence as described previously (Wang *et al.*, 2011). Cells stained with LC3 (green) and nuclei (blue) were analyzed using an LSM 510 confocal microscope (Carl Zeiss, Oberkochen, Germany). In each treatment group, the average LC3 puncta per cell was calculated by analyzing at least 200 cells.

### 2.6 Luciferase reporter assay

The 3′UTR sequence of human DANCR was cloned from the downstream of pGL3 luciferase reporter vector (Promega) to generate DANCR luciferase reporter plasmid (pGL3‐DANCR‐wt). The 3′UTR sequence of human DANCR containing mutation sites for miR‐874‐3P was generated using the QuikChange II XL Site‐Directed Mutagenesis Kit (Stratagene, La Jolla, CA, USA) to construct pGL3‐DANCR‐mut plasmid. The plasmids of pGL3‐ATG16L1‐wt and pGL3‐ATG16L1‐mut containing mutation sites for miR‐874‐3P were developed as described above. The sequence of all plasmids was confirmed by sequencing. Luciferase reporter plasmids were cotransfected with miR‐874‐3P mimics or miR‐NC into HL60 cells *via* the FuGENE 6 Transfection Reagent (Promega) according to the manufacturer's instructions. After 48 h of transfection, luciferase activity was measured using the Dual‐Luciferase Reporter Assay System (Promega). Data were normalized to the results of Renilla luciferase activity.

### 2.7 RNA pull‐down assay

The DANCR pull‐down assay was performed as previously described (Yuan *et al.*, 2014). Briefly, DANCR‐wt and DANCR‐mut (mutant for miR‐874‐3P) were transcribed *in vitro* from vector pSPT19‐DANCR. Transcripts were then labeled with biotin using the Biotin RNA Labeling Mix (Roche, Indianapolis, IN, USA) and T7 RNA polymerase (Roche), digested with RNase‐free DNase I (Roche), and finally purified with the RNeasy Mini Kit (Qiagen, Hilden, Germany) according to the manufacturer's instructions. The whole‐cell lysates of HL60 cells were incubated for 1 h with 2 µg purified biotinylated transcripts at 25 °C, and complexes were isolated using streptavidin agarose beads (Invitrogen). The level of miR‐874‐3P, miR‐634, and miR‐496 in the pull‐down products of biotin‐labeled DANCR or empty beads was quantified by qRT‐PCR analysis.

### 2.8 RNA immunoprecipitation assay

Anti‐AGO2 RNA immunoprecipitation (RIP) assay was conducted as documented before with the Magna RIP RNA‐Binding Protein Immunoprecipitation Kit (Millipore, 17‐700) following the manufacturer's instructions (Yuan *et al.*, 2014). In brief, HL60 cells were lysed in RIP lysis buffer, and the whole‐cell lysates were incubated with protein A/G magnetic beads conjugated with human anti‐AGO2 antibody or normal mouse IgG antibody. After rinsing, the binding RNA was retrieved and then determined by qRT‐PCR analysis. U6 was used as a nontarget control.

### 2.9 Statistical analysis

All data are expressed as means ± standard deviation (SD). Statistical analysis was performed using spss 17.0 software (SPSS, Chicago, IL, USA), and Student's *t*‐test was applied to calculate the significance when comparing two sets of data. *P* values < 0.01 or 0.05 were considered statistically significant.

## 3 Results

### 3.1 DANCR expression is elevated in human AML cells treated with Ara‐C

**Fig. 1:**
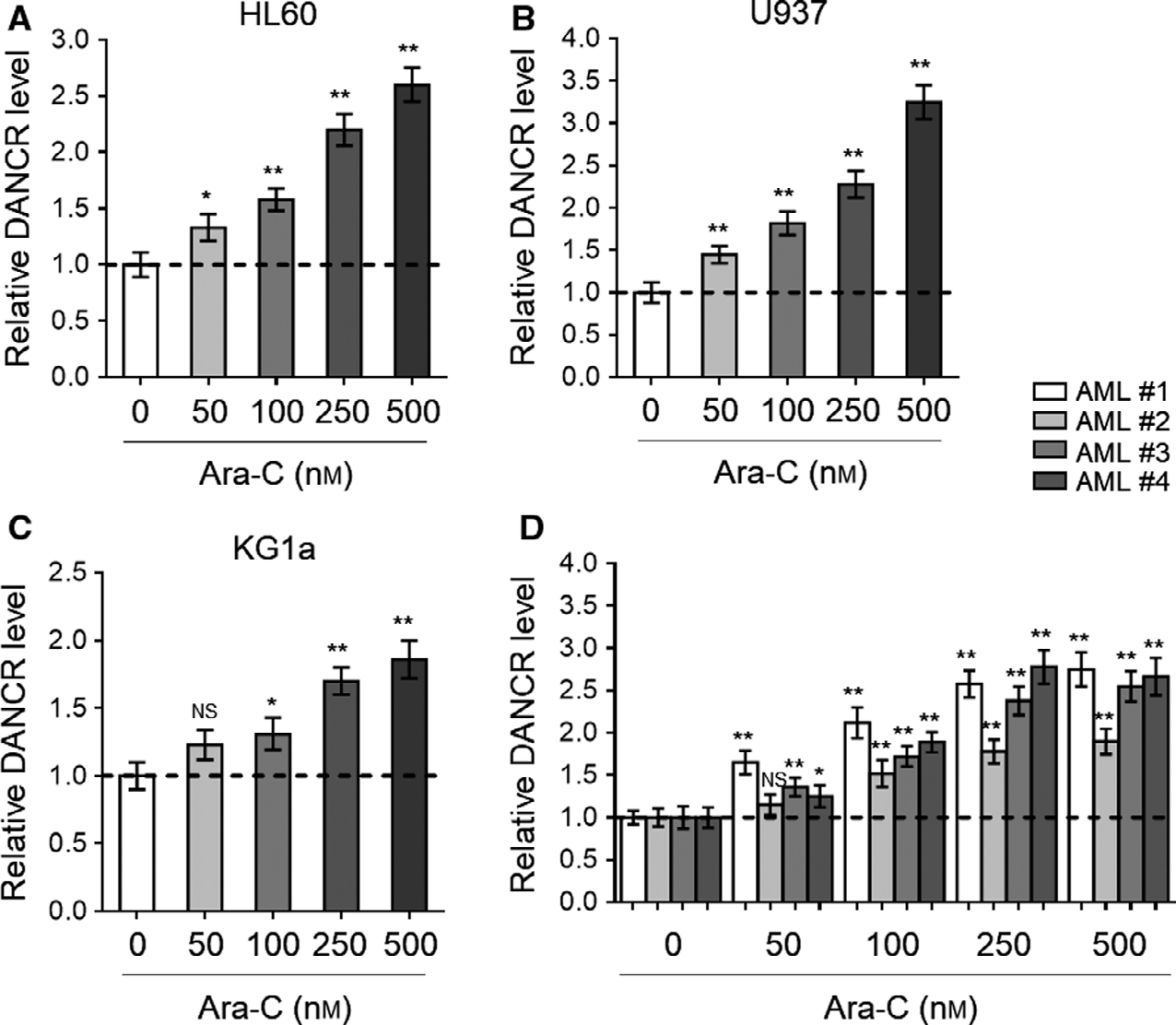
Ara‐C treatment elevates DANCR expression in human AML cells. (A–D) Human AML cell line HL60 (A), U937 (B), and KG1a (C) and four lines of primary AML cells from newly diagnosed patients (denoted as AML #1, AML #2, AML #3, and AML #4) (D) were treated with increasing concentrations of Ara‐C as indicated for 24 h. The expression of DANCR was determined by qRT‐PCR analysis. β‐Actin was used as an endogenous control. The results are expressed as relative to vehicle treatment. Each column represents the value from three replicates. All data are mean ± SD. Data were analyzed using Student's *t*‐test. **P < 0.01; *P < 0.05; NS, not significant.

The high expression of DANCR has been linked with tumor progression and poor prognosis in colorectal cancer (Liu *et al.*, 2015), hepatocellular carcinoma (Ma *et al.*, 2016), and gastric cancer (Hao *et al.*, 2017). Besides, the highly expressed DANCR is also associated with the frequency of AML stem cells (Bill *et al.*, 2018). Furthermore, intriguingly, DANCR expression is negatively correlated with cisplatin sensitivity in glioma cells (Ma *et al.*, 2018). These studies hint that DANCR may function as an oncogenic lncRNA involved in the regulation of drug resistance. To explore whether DANCR is associated with Ara‐C resistance in AML, we first asked whether DANCR expression may respond to Ara‐C treatment in human AML cells. To test this possibility, we treated three human AML cell lines, including HL60, U937, and KG1a, with escalating dosage of Ara‐C (ranging from 0 to 500 nm), and then checked DANCR expression *via* qRT‐PCR analysis. The results showed that DANCR expression was dose‐dependently elevated in HL60 (Fig. 1A), U937 (Fig. 1B), and KG1a (Fig. 1C) cells when treated with Ara‐C. Moreover, similar results were obtained when four primary AML cell lines, sampled from newly diagnosed pediatric AML patients, were treated with Ara‐C (Fig. 1D). Two previous studies have shown that DANCR is a direct transcriptional target of Sox4 (Zhang *et al.*, 2015) and Sall4 (Pan *et al.*, 2018). However, in HL‐60 cells, compared with siRNA control (siCtrl), the depletion of Sox4 (Fig. S1A) and Sall4 (Fig. S1B) had no obvious effects on Ara‐C‐induced DANCR expression, indicating that these two transcriptional factors are irrelevant to DANCR regulation in response to Ara‐C treatment. Together, these results suggest that Ara‐C treatment induces DANCR expression in human AML cells, at least under an *in vitro* experimental condition.

### 3.2 DANCR confers Ara‐C resistance in human AML cells

**Fig. 2:**
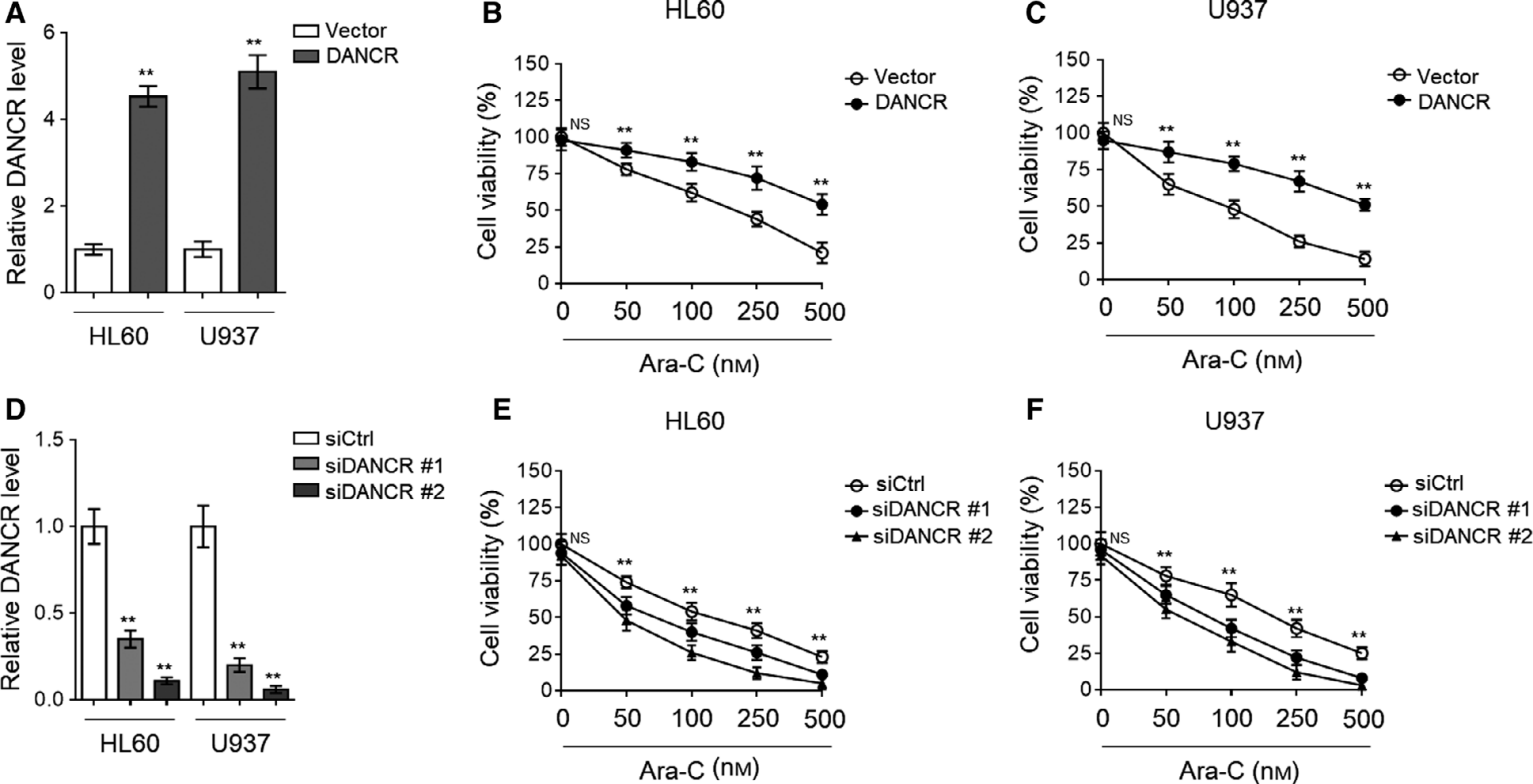
DANCR increases Ara‐C resistance in human AML cells. (A–C) HL60 and U937 cells were transfected with empty vector pcDNA3.1 or pcDNA3.1 expressing DANCR for 48 h, and then treated with increasing concentrations of Ara‐C as indicated for 24 h. (A) The level of DANCR was determined by qRT‐PCR analysis. (B, C) Cell viability of HL60 (B) and U937 (C) cells was analyzed by MTT assay. The results are expressed as percentage of vehicle treatment (%). (D, F) HL60 and U937 cells were transfected with siRNA targeting scrambled sequence (siCtrl) or DANCR (siDANCR #1 or siDANCR #2) for 48 h, and then treated with increasing concentrations of Ara‐C as indicated for 24 h. (D) The level of DANCR was determined by qRT‐PCR analysis. (E, F) Cell viability of HL60 (E) and U937 (F) cells was analyzed by MTT assay. The results are expressed as percentage of vehicle treatment (%). Each symbol represents the value from five replicates. All data are mean ± SD. Data were analyzed using Student's *t*‐test. **P < 0.01; NS, not significant.

Since DANCR expression responds to Ara‐C treatment by upregulation in AML cells, we next asked whether DANCR plays a functional role in Ara‐C resistance. To address this issue, we transiently overexpressed DANCR in HL60 and U937 cells, and then tested the influence on cell viability under the treatment of Ara‐C. Firstly, the effective overexpression of DANCR in both HL60 and U937 cells was confirmed by qRT‐PCR analysis, as compared to vector control (Fig. 2A). Then, MTT assay showed that under the treatment of escalating dosage of Ara‐C, the cell viability of both HL60 (Fig. 2B) and U937 (Fig. 2C) was significantly increased when DANCR was overexpressed. To consolidate the role of DANCR in affecting Ara‐C resistance in AML cells, conversely, we employed siRNA technique to deplete DANCR expression in HL60 and U937 cells. Two specific siRNAs with different sequences targeting DANCR (siDANCR #1 and siDANCR #2) were utilized, and compared with siRNA control (siCtrl), they both showed remarkable effect to silence DANCR expression in HL60 and U937 cells, of note, with siDANCR #2 being more effective (Fig. 2D). Furthermore, just contrary to those findings observed with DANCR overexpression (Fig. 2B,C), DANCR depletion significantly reduced cell viability of HL60 (Fig. 2E) and U937 (Fig. 2F) cells treated with Ara‐C. Notably, compared with siDANCR #1, the cell viability of HL60 and U937 cells transfected with siDANCR #2 was obviously lower (Fig. 2E,F), strengthening a correlation between DANCR expression and chemosensitivity to Ara‐C in AML cells. Collectively, these results suggest that DANCR reduces the chemosensitivity of AML cells to Ara‐C treatment, or in other words, DANCR functions to confer Ara‐C resistance in AML cells.

### 3.3 DANCR promotes autophagy in HL60 cells treated with Ara‐C

**Fig. 3:**
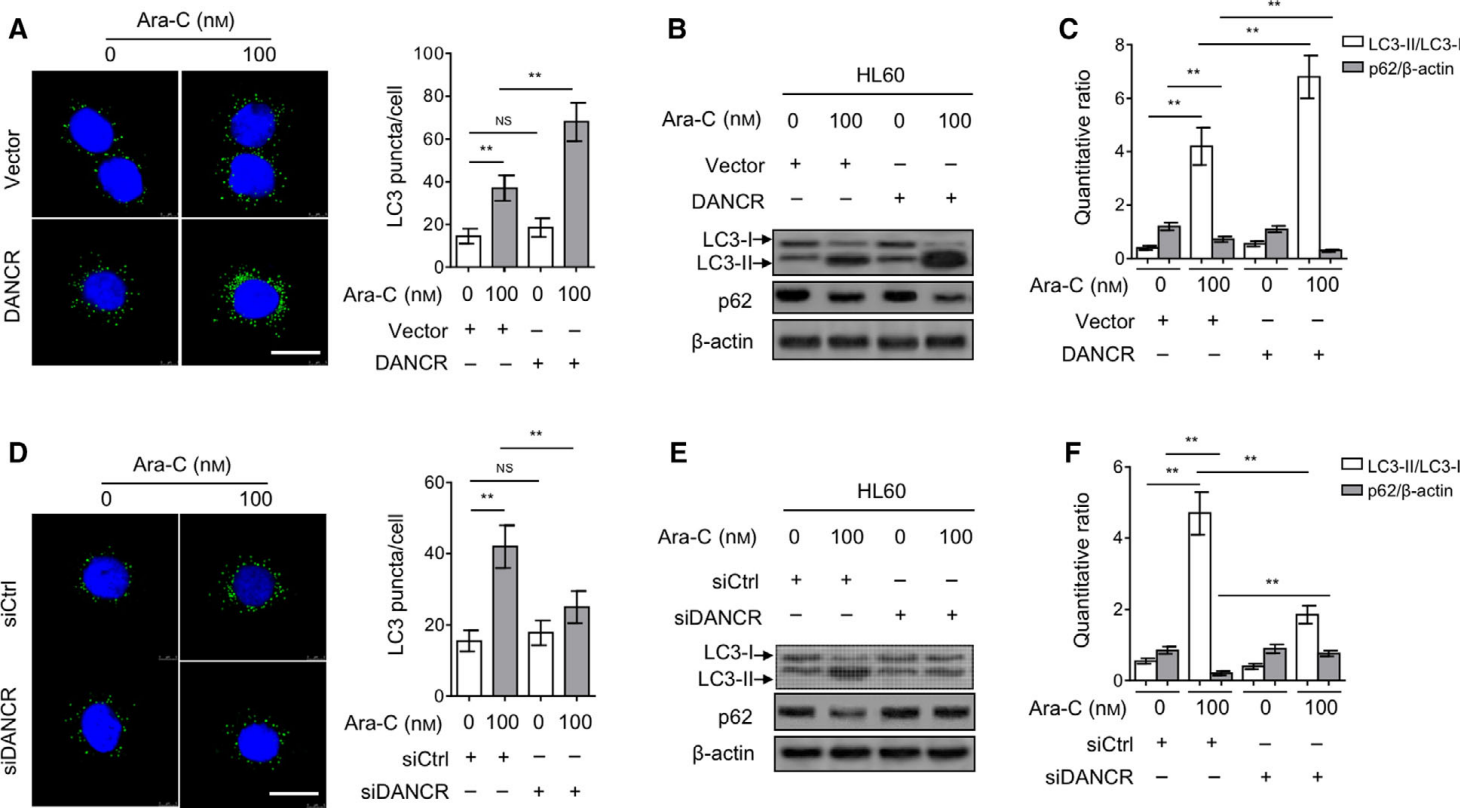
DANCR promotes Ara‐C‐induced autophagy in HL60 cells. (A–C) HL60 cells were transfected with empty vector pcDNA3.1 or pcDNA3.1 expressing DANCR for 48 h, and then treated with or without 100 nm Ara‐C for 24 h. (A) The LC3 puncta was visualized by immunofluorescence assay. The representative cells from two independent experiments are shown. Green, LC3 puncta; blue, nuclei. Scale bar, 20 µm. (B, C) The protein expression of LC3 and p62 was measured by immunoblotting. β‐Actin was used as a loading control. The representative images (B) and ratio of LC3‐II/LC3‐I and p62/β‐actin (C) are shown. (D–F) HL60 cells were transfected with siRNA targeting scrambled sequence (siCtrl) or DANCR (siDANCR #2) for 48 h, and then treated with or without 100 nm Ara‐C for 24 h. The LC‐3 puncta (D), and protein expression of LC‐3 and p62 (E, F) were analyzed as in (A–C). All data are expressed as mean ± SD. Data were analyzed using Student′s *t*‐test. **P < 0.01; NS, not significant.

Some studies have shown that autophagy plays a role in conferring Ara‐C resistance in AML cells (Auberger and Puissant, 2017; Bosnjak *et al.*, 2014; Piya *et al.*, 2017). To elucidate the mechanism by which DANCR confers Ara‐C resistance in AML cells, we next examined whether DANCR is able to modulate autophagy. Lysosomal turnover of LC3‐II is a specific marker for autophagy activity (Tanida *et al.*, 2005). Coinciding with a previous report (Bosnjak *et al.*, 2014), we found that Ara‐C treatment induced autophagy in HL60 cells, as shown by increased number of LC3 puncta (Fig. 3A), accelerated turnover of LC3‐II, and promoted degradation of autophagic substrate sequestosome 1 (SQSTM1/p62; Fig. 3B,C, lane 1 vs. lane 2). Moreover, under Ara‐C treatment, DANCR‐overexpressing HL60 cells showed a higher autophagic induction than those transfected with vector control (Fig. 3A, right half; Fig. 3B,C, lane 2 vs. lane 4); however, DANCR overexpression alone could not obviously affect autophagy in HL60 cells (Fig. 3A, left half; Fig. 3B,C, lane 1 vs. lane 3). These results suggest that DANCR promotes Ara‐C‐induced autophagy in HL60 cells. Additionally, this notion was further confirmed by the evidence showing that DANCR depletion through siRNA drastically diminished Ara‐C‐induced autophagy in HL60 cells (Fig. 3D–F). Taken together, these data show that autophagy activity is promoted by DANCR in Ara‐C‐treated AML cells, which might be a relevant event for Ara‐C resistance conferred by DANCR.

### 3.4 DANCR directly targets miR‐874‐3P in AML cells

**Fig. 4:**
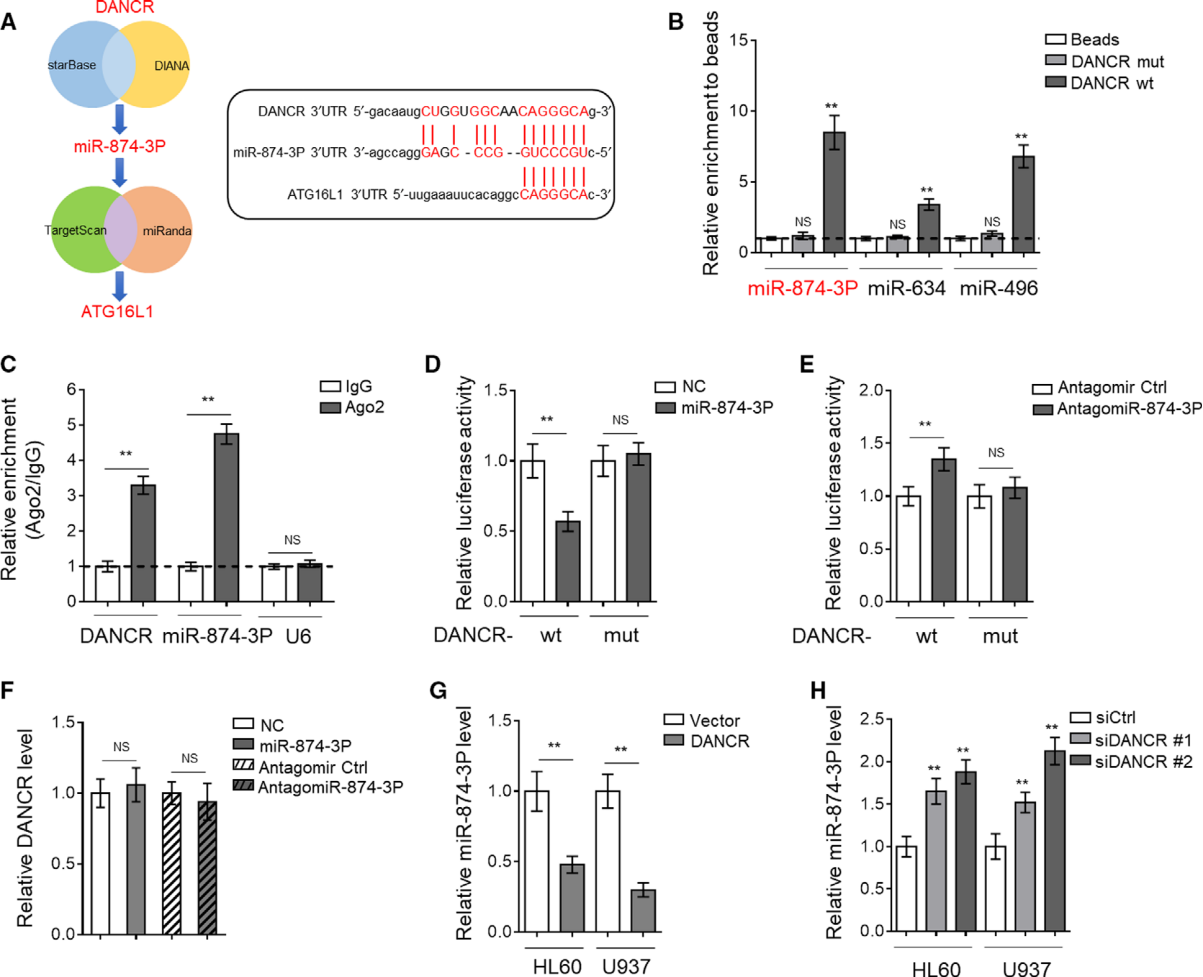
DANCR acts as a sponge of miR‐874‐3P. (A) The Venn diagram (left) of identifying miR‐874‐3P as a common miRNA targeting both DANCR 3′UTR and ATG16L1 3′UTR, and bioinformatic prediction (right) of miR‐874‐3P binding sites in DANCR 3′UTR sequence using StarBase or ATG16L1 3′UTR sequence using TargetScan. (B) DANCR biotin pull‐down assay was performed using lysates of HL60 cells. The level of target miR‐874‐3P, miR‐634, and miR‐496 in the pull‐down products of biotin‐labeled wt DANCR or mutant DANCR (mut) was quantified by qRT‐PCR analysis. The results are expressed as a fold enrichment of empty beads. (C) RIP assay was conducted to detect the enrichment of DANCR and miR‐874‐3P by using Ago2 antibody and lysates of HL60 cells. Isotype IgG was used as an internal antibody control. U6 was used as a nontarget control. The results are expressed as a fold enrichment of IgG control. (D) HL60 cells were cotransfected with pMIR‐LUC‐3′‐UTR‐DANCR‐wt (DANCR‐wt) or DANCR‐mut with 100 nm O/E miR‐874‐3P or 100 nm O/E NC for 48 h, and then, luciferase activity was measured. The results relative to NC group are shown. (E) HL60 cells were cotransfected with DANCR‐wt or DANCR‐mut with 100 nm antagomir Ctrl or 100 nm antagomiR‐874‐3P for 48 h, and then, luciferase activity was measured. The results relative to antagomir Ctrl group are shown. (F) HL60 cells were treated as in (D, E). The expression of DANCR was determined by qRT‐PCR analysis. β‐Actin was used as an endogenous control. (G, H) HL60 and U937 cells were transfected with empty pcDNA3.1 vector or pcDNA3.1 expressing DANCR (G) or siRNA targeting scrambled sequence (siCtrl) or DANCR (siDANCR #1 or siDANCR #2) (H) for 48 h. The expression of miR‐874‐3P was determined by qRT‐PCR analysis. β‐Actin was used as an endogenous control. All data are expressed as mean ± SD. *n* = 3. Data were analyzed using Student's *t*‐test. **P < 0.01; NS, not significant.

Long noncoding RNAs are known to protect mRNAs through acting as competing endogenous RNAs (ceRNAs) to sponge their targeted miRNAs (Tay *et al.*, 2014). Besides, miRNAs play an important role in regulating autophagy in cancer cells (Frankel and Lund, 2012). Thus, to check whether DANCR regulates autophagy through sponging autophagy‐regulating miRNAs, we applied bioinformatic analysis to search potential miRNAs that target both the DANCR 3′UTR (DIANA and StarBase v2.0) and the 3′UTR of genes involved in autophagy process (TargetScan and miRanda; Fig. 4A, left). Through prediction, we found that DANCR and ATG16L1 share a common binding sequence in 3′UTR of miR‐874‐3P (Fig. 4A, right), implying that DANCR may bind with miR‐874‐3P, which possibly targets ATG16L1, one key autophagy gene embedded in autophagy paradigm (Cadwell *et al.*, 2008). We first tested whether DANCR could directly interact with miR‐874‐3P *via* RNA pull‐down assay using biotin‐labeled DANCR and whole‐cell lysates of HL60. Indeed, as shown in Fig. 4B, DANCR with wild‐type 3′UTR sequence (wt) interacted with miR‐874‐3P, and DANCR with mutations (mut) in miR‐874‐3P targeting sites had no similar effect. As positive controls, miR‐634 and miR‐496, two miRNAs previously shown to bind with DANCR (Lu *et al.*, 2018; Xu *et al.*, 2018), were simultaneously found to present in pull‐down products, confirming the validity of this assay. Moreover, the specific interaction between DANCR and miR‐874‐3P was also supported by the evidence obtained from anti‐Ago2 RIP assay (Fig. 4C).

For further confirmation, we constructed luciferase reporters containing the 3′UTR sequence of DANCR with wt or mutated miR‐874‐3P binding sites (mut). We found that compared with negative control (NC) overexpression, miR‐874‐3P overexpression reduced the luciferase activity of DANCR‐wt reporter vector but not mut reporter vector (Fig. 4D). Conversely, miR‐874‐3P knockdown *via* antagomiR‐874‐3P transfection increased the luciferase activity of DANCR‐wt reporter vector but not mut reporter vector, as compared to antagomir control (Fig. 4E). But no significant difference in DANCR expression was observed in HL60 cells after either overexpression or knockdown of miR‐874‐3P (Fig. 4F). Hence, these data indicate that miR‐874‐3P interacts with DANCR but does not cause its degradation. However, oppositely, DANCR overexpression resulted in decreased miR‐874‐3P level (Fig. 4G) and its knockdown elevated (Fig. 4H) miR‐874‐3P level in both HL60 and U937 cells, suggesting that DANCR directly targets and sponges miR‐874‐3P, which results in the decreased level of miR‐874‐3P in AML cells.

### 3.5 DANCR induces ATG16L1 expression through sponging miR‐874‐3P in AML cells

**Fig. 5:**
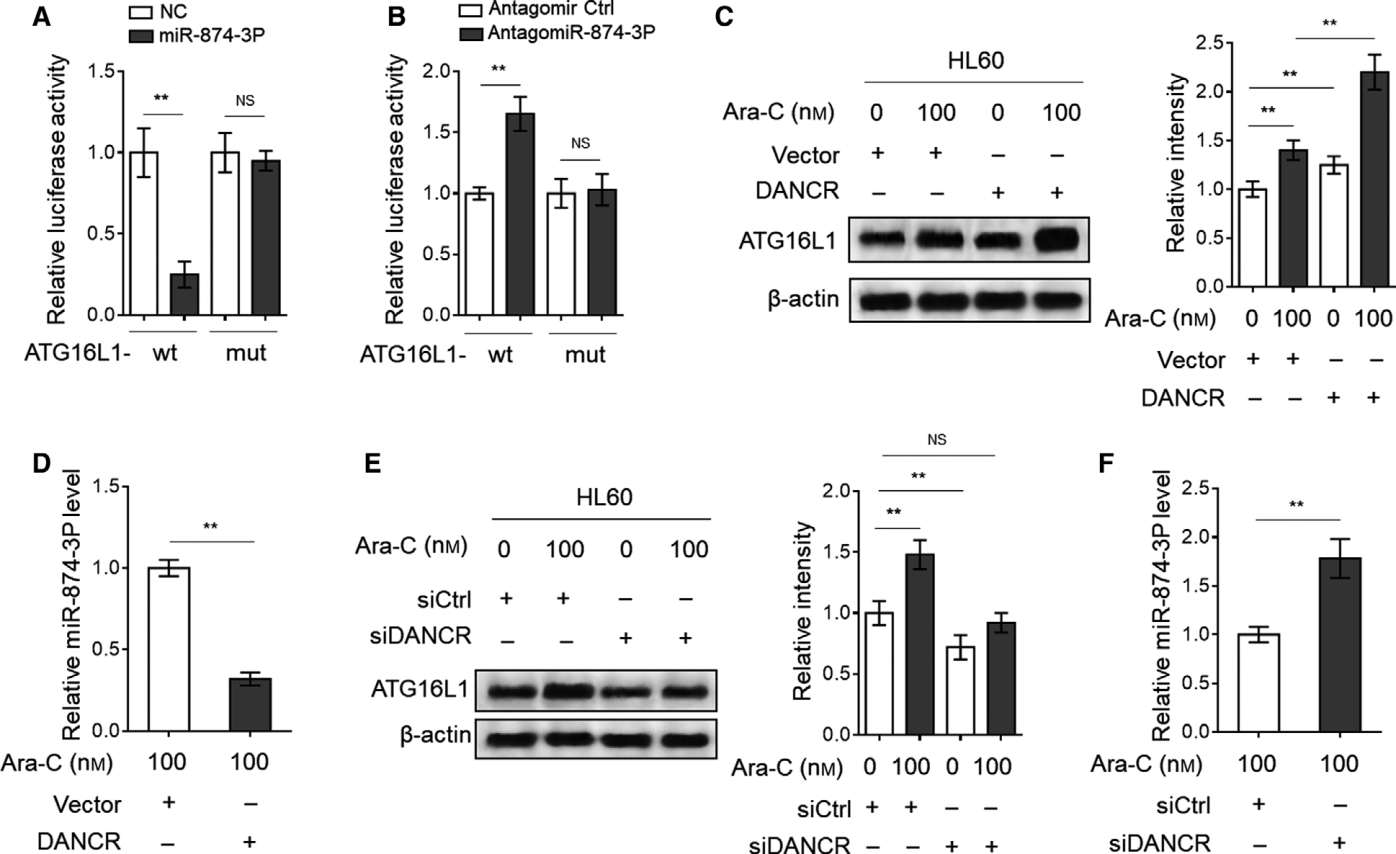
DANCR induces ATG16L1 expression in AML cells through sponging miR‐874‐3P. (A) HL60 cells were cotransfected with pMIR‐LUC‐3′‐UTR‐ATG16L1‐wt (ATG16L1‐wt) or ATG16L1‐mut with 100 nm O/E miR‐874‐3P or 100 nm O/E NC for 48 h, and then, luciferase activity was measured. The results relative to NC group are shown. (B) HL60 cells were cotransfected with ATG16L1‐wt or ATG16L1‐mut with 100 nm antagomir Ctrl or 100 nm antagomiR‐874‐3P for 48 h, and then, luciferase activity was measured. The results relative to antagomir Ctrl group are shown. (C, D) HL60 cells were transfected with empty vector pcDNA3.1 or pcDNA3.1 expressing DANCR for 48 h, and then treated with or without 100 nm Ara‐C for 24 h. The expression of ATG16L1 (C) and miR‐874‐3P (D) was measured by immunoblotting and qRT‐PCR, respectively. The band intensity analysis of ATG16L1 is also shown. (E, F) HL60 cells were transfected with siRNA targeting scrambled sequence (siCtrl) or DANCR (siDANCR #2) for 48 h, and then treated with or without 100 nm Ara‐C for 24 h. The expression of ATG16L1 (E) and miR‐874‐3P (F) was measured by immunoblotting and qRT‐PCR, respectively. The band intensity analysis of ATG16L1 is also shown. All data are expressed as mean ± SD. *n* = 3. Data were analyzed using Student's *t*‐test. **P < 0.01; NS, not significant.

Since we have demonstrated that DANCR acts to sponge miR‐874‐3P to establish a possible causal link between DANCR and autophagy regulation, we examined whether miR‐874‐3P directly targets ATG16L1, as predicted by TargetScan and miRanda (Fig. 4A). To this end, we constructed luciferase reporters containing the 3′UTR sequence of ATG16L1 with wt or mutated miR‐874‐3P binding sites (mut). Luciferase reporter assay showed that in HL60 cells, overexpression of miR‐874‐3P (Fig. 5A) and its knockdown (Fig. 5B) respectively reduced and increased the luciferase activity of ATG16L1 wt reporter vector but not mut reporter vector, proving that ATG16L1 is a direct target of miR‐874‐3P in HL60 cells. This finding led us to speculate that DANCR may positively regulate ATG16L1 expression through sponging miR‐874‐3P. Expectedly, we found that DANCR overexpression not only significantly increased ATG16L1 expression in HL60 cells without Ara‐C treatment, but also further increased Ara‐C‐induced expression of ATG16L1 under the treatment of Ara‐C (Fig. 5C). Consistent with the negative regulation of DANCR in miR‐874‐3P expression (Fig. 4G), DANCR overexpression resulted in decreased level of miR‐874‐3P in Ara‐C‐treated HL60 cells (Fig. 5D). Given that miR‐874‐3P directly targets ATG16L1 (Fig. 5A,B), in any case, these results conclude that DANCR induces ATG16L1 expression through sponging miR‐874‐3P in HL60 cells. This conclusion was further supported by the findings that DANCR knockdown abolished Ara‐C‐induced expression of ATG16L1 (Fig. 5E), and, meanwhile, increased miR‐874‐3P level in Ara‐C‐treated HL60 cells (Fig. 5F). Of note, both DANCR overexpression (Fig. S2A) and knockdown (Fig. S2B) had no obvious effects on the expression of Beclin‐1, ATG5, and ATG7, three important autophagy‐related proteins (Levine and Klionsky, 2004), which suggests a specificity of DANCR in targeting autophagy machinery. Based on these results, DANCR may act as a regulator of Ara‐C‐induced autophagy through miR‐874‐3P/ATG16L1 axis in AML cells.

### 3.6 ATG16L1 silencing abrogates DANCR‐promoted autophagy and diminishes Ara‐C resistance in AML cells

**Fig. 6:**
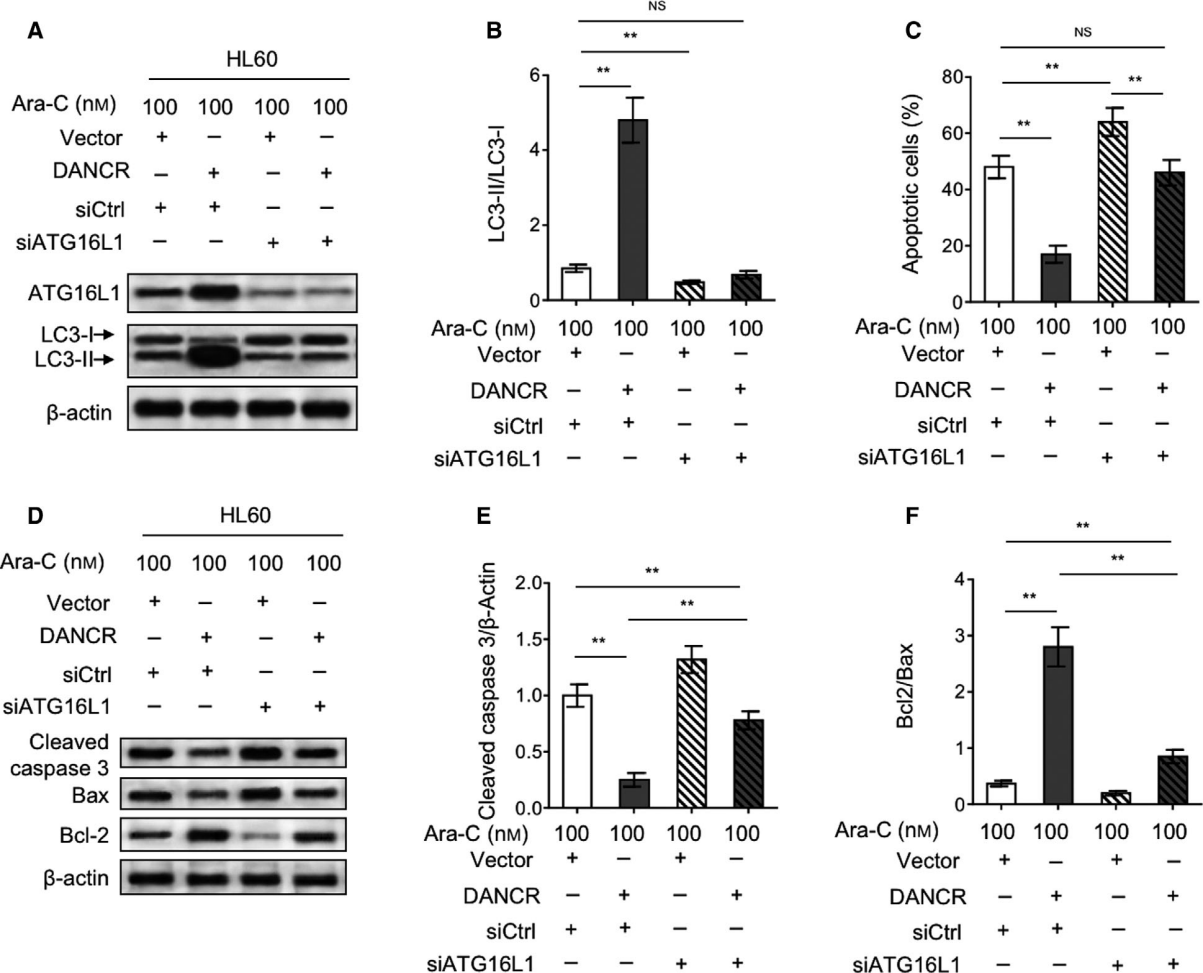
ATG16L1 silencing abrogates DANCR‐promoted autophagy and markedly recovers DANCR‐conferred Ara‐C resistance. (A, B) HL60 cells were cotransfected with empty vector pcDNA3.1 or pcDNA3.1 expressing DACR with siRNA targeting scrambled sequence (siCtrl) or ATG16L1 (siATG16L1) for 48 h in the presence of 100 nm Ara‐C. The protein expression of ATG16L1 and LC‐3 was measured by immunoblotting. β‐Actin was used as a loading control. The representative images (A) and statistical analysis of LC3‐II/LC3‐I (B) are shown. (C) HL60 cells were treated as in (A, B). Cell apoptosis was analyzed by annexin V/PI staining assay. The results are expressed as percentage of annexin V‐positive cells among total cells (%). (D–F) HL60 cells were treated as in (A, B). The protein expression of cleaved caspase‐3, Bax, and Bcl‐2 was measured by immunoblotting. β‐Actin was used as a loading control. The representative images (D) and statistical analysis of the fold change of cleaved caspase‐3 (E) and Bax/Bcl‐2 (F) are shown. Data are mean ± SD and compared using Student's *t*‐test. *n* = 3. **P < 0.01; NS, not significant.

At last, to clarify the role of ATG16L1 in DANCR‐promoted autophagy and Ara‐C resistance in AML cells, we silenced ATG16L1 expression *via* siRNA transfection in Ara‐C‐treated HL60 cells with or without DANCR overexpression (Fig. 6A). Supporting the importance of ATG16L1 in autophagy activation (Ishibashi *et al.*, 2012; Salem *et al.*, 2015), ATG16L1 silencing not only reduced the basal level of autophagy under Ara‐C treatment (Fig. 6A,B, lane 1 vs. lane 3), but also totally abrogated DANCR‐promoted autophagy in HL60 cells, resulting in a recovery to that of vector group, as shown by the turnover of LC3‐II (Fig. 6A,B, lane 1 vs. lane 4). Functionally, along with the inhibition of autophagy through ATG16L1 silencing, HL60 cell viability was decreased (Fig. S3A, column 1 vs. column 3) and apoptosis was increased (Fig. 6C, column 1 vs. column 3) under the treatment of Ara‐C, which points to a protective role of autophagy induction in antagonizing the cytotoxicity of Ara‐C. More importantly, DANCR overexpression‐increased cell viability (Fig. S3A, column 1 vs. column 2) and DANCR overexpression‐decreased apoptosis (Fig. 6C, column 1 vs. column 2) were all statistically recovered to those of the vector group (column 1 vs. column 4), although they still exhibited significant change in DANCR‐overexpressing group when ATG16L1 was silenced (column 3 vs. column 4). Furthermore, similar results were obtained when HL60 cells were treated with bafilomycin A1 (Fig. S3B) and chloroquine (Fig. S3C), two widely used autophagy inhibitors (Redmann *et al.*, 2017). These data suggest that the ATG16L1‐regulated autophagy formation plays a critical role in mediating DANCR‐conferred Ara‐C resistance in AML cells and that other mechanisms underlying DANCR effect on Ara‐C resistance could not be ruled out.

**Fig. 7:**
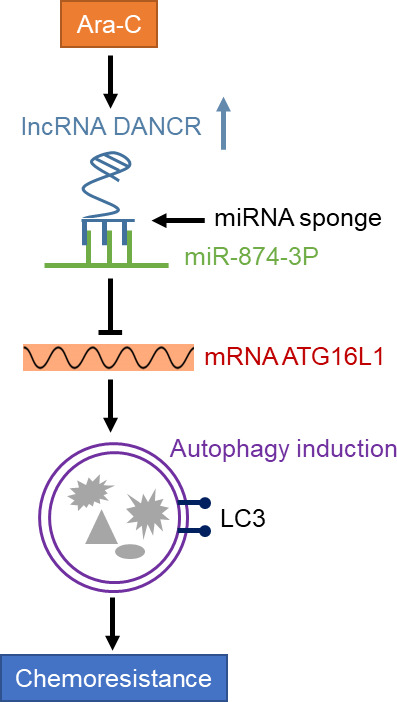
A schematic model of DANCR in enhancing Ara‐C resistance in AML cells. Ara‐C induces the expression of DANCR in AML cells, which acts as a ceRNA to directly bind and sponge miR‐874‐3P, resulting in the release of inhibition on ATG16L1 mRNA and induction of autophagy that in turn mediates the chemoresistance to Ara‐C.

The cytotoxicity of Ara‐C in AML cells could be largely reflected by the activation of apoptotic pathway (Adams *et al.*, 2017). In accordance with the improved cell viability, DANCR overexpression decreased the level of cleaved caspase‐3 (Fig. 6D,E, column 1 vs. column 2) and increased the ratio of Bcl2/Bax (Fig. 6D,F, column 1 vs. column 2), two opposite indicators of apoptosis, in HL60 cells treated with Ara‐C. However, when ATG16L1 was silenced, the alteration of cleaved caspase‐3 and the ratio of Bcl2/Bax was remarkably recovered (Fig. 6D,F, column 2 vs. column 4), although not completely reached to those of vector control (Fig. 6D,F, column 1 vs. column 4). Nonetheless, together with evidence shown in Fig. 6A–C, these results undoubtedly indicate that ATG16L1‐mediated autophagy exerts an antiapoptotic role against the cytotoxicity of Ara‐C, which constitutes a key explanation for the DANCR‐conferred Ara‐C resistance in AML cells. In summary, these lines of evidence identify the miR‐874‐3P/ATG16L1 axis as an underlying mechanism of the DANCR‐regulated autophagy that mediates the promotive effect of DANCR on Ara‐C resistance in AML cells (Fig. 7).

## 4 Discussion

With the advent of next‐generation sequencing, a growing number of lncRNAs have been exposed from the cancer transcriptomes (Prensner and Chinnaiyan, 2011). Subsequent functional studies have demonstrated that some lncRNAs play a key role in gene regulation and their aberrant expression is linked to malignant transformation through affecting various aspects of cellular activities, including proliferation, survival, migration, and genomic stability (Huarte, 2015). Among these lncRNAs, DANCR emerges as a promising tumor‐associated lncRNA and its aberrant expression was found to be of certain clinical significance in various cancers through considerable association with augmented typical cancer hallmarks such as cell proliferation, stemness, invasion, and metastasis (Thin *et al.*, 2018). Due to these oncogenic roles, as one of the critical regulatory RNAs in human cancers, DANCR was proposed to be a potential therapeutic target that holds a great promise for future cancer therapy (Thin *et al.*, 2018). However, to date, the role of DANCR in AML is less known, except for one previous study reporting that DANCR regulates the function of AML stem cells (Bill *et al.*, 2018). In the present study, we aimed to explore the association between DANCR and Ara‐C resistance in AML, one clinical challenge hindering effective AML chemotherapy (Schneider *et al.*, 2017). We initially found that DANCR expression was induced in AML cell lines and primary AML cells in response to Ara‐C treatment. The following functional and mechanistic studies show that DANCR is able to enhance the chemoresistance of AML cells to Ara‐C treatment through an autophagy‐dependent manner *via* sponging miR‐874‐3P/ATG16L1 axis, since DANCR promotes Ara‐C‐induced autophagy by increasing ATG16L1 level through directly interacting with miR‐874‐3P and reducing its level, and moreover, autophagy inhibition *via* ATG16L1 depletion markedly diminishes DANCR‐elicited chemoresistance in AML cells (Fig. 7). Therefore, our study may shed new light on the role and mechanism through which DANCR could exert its oncogenic activity in AML.

To our knowledge, it is the first time the induction of DANCR expression is linked to Ara‐C treatment in AML cells, probably uncovering it as an Ara‐C‐responsive lncRNA. We have provided evidence demonstrating that the upregulation of DANCR induced by Ara‐C is independent on Sox4 and Sall4. Currently, how Ara‐C treatment increases DANCR expression at a molecular level remains unclear. Further studies are required to address this issue. Through gain‐of‐function and loss‐of‐function approaches, DANCR was found to increase the chemoresistance of AML cells to Ara‐C. It appears that the induction of DANCR expression in AML cells may be triggered as a prosurvival adaptive response for counteracting the cytotoxicity of Ara‐C. The expression profile of lncRNAs is associated with clinical features and outcome in AML patients (Garzon *et al.*, 2014). In two RNA‐seq datasets reported recently, DANCR was identified to be correlated strongly with features of leukemia stem cells, a subgroup of cells showing increased chemotherapy resistance (Bill *et al.*, 2019). Therefore, it might be of interests to test whether DANCR expression is associated with Ara‐C resistance in AML patients and able to predict prognosis after chemotherapy. In fact, except for increasing Ara‐C resistance in AML cells, DANCR has also been shown to mediate cisplatin resistance in glioma cells (Ma *et al.*, 2018), implying that DANCR may be implicated in the regulation of chemoresistance in other scenarios. Future efforts focused on investigating the functional role of DANCR in chemoresistance could help to broaden our knowledge on cancer‐associated activities of DANCR.

One critical mechanism by which lncRNAs regulate gene expression is acting as ceRNAs to sponge targeted miRNAs (Salmena *et al.*, 2011). This is also true for DANCR, not only because several miRNAs have been identified as direct targets of DANCR, but also because the function of DANCR is prominently mediated through downregulating the expression of these miRNAs, as reported by recent studies (Liang *et al.*, 2019; Lu *et al.*, 2018; Wang *et al.*, 2018; Wang *et al.*, 2019; Xu *et al.*, 2018; Yang *et al.*, 2018), to name a few. Combining predictive methods and some biochemical experiments, we demonstrate that miR‐874‐3P is a new binding target of DANCR and that by suppressing the inhibitory effect of miR‐874‐3P on ATG16L1 expression *via* interacting and decreasing miR‐874‐3P level, DANCR possesses an ability to upregulate ATG16L1 expression. In theory, due to this regulation, the expression of DANCR would be positively correlated with that of ATG16L1. Indeed, we noticed that DANCR increased ATG16L1 expression in AML cells under both treated and untreated conditions (Fig. 5C). However, noteworthily, this tendency is not faithfully consistent with the effect of DANCR on autophagy activity, as we observed that DANCR that only promoted Ara‐C‐induced autophagy in AML cells, however, had no similar effect on the basal level of autophagy (Fig. 3). We speculate that ATG16L1 may not be indispensable for maintaining the physiological level of autophagy in AML cells, but under the treatment of Ara‐C, more amount of ATG16L1 is required to support the induction of autophagy. The negative regulation of miR‐874‐3P on ATG16L1 was also shown to result in suppressed autophagy when induced by rapamycin (Yamaguchi *et al.*, 2018). Furthermore, ATG16L1‐mediated autophagy is involved in multiple‐drug resistance in gastric cancer (Huang *et al.*, 2018), together with our findings, suggesting that the miR‐874‐3P/ATG16L1 axis‐regulated autophagy may be an important tactic through which cancer cells utilize to survive the insults of chemotherapeutic drugs. Further, although ATG16L1 knockdown and treatment of autophagy inhibitors totally reverse the DANCR‐increased Ara‐C resistance to the level of vector group, DANCR overexpression still results in higher level of Ara‐C resistance under these conditions (Fig. 6C and Fig. S3), which implies that other mechanisms may also account for this phenomenon. Fully addressing this issue is helpful to advance our understanding of how DANCR increases Ara‐C resistance in AML cells.

## 5 Conclusions

In summary, we identify DANCR as a novel positive regulator of Ara‐C resistance in AML cells, in which the enhanced autophagy through modulating the miR‐874‐3P/ATG16L1 axis is the key molecular event that plays an antiapoptotic role against Ara‐C cytotoxicity. Future investigations using xenografted tumor model with nude mice would bring more profound significance by investigating whether DANCR blocking reverses Ara‐C resistance in AML cell. Based on the findings shown in the present study, we propose here that DANCR may be considered as a possible biomarker for predicting Ara‐C resistance and a therapeutic target for improving the treatment efficacy of Ara‐C in AML.

## Conflict of interest

The authors declare no conflict of interest.

## Author contributions

HZ performed the experiments and analyzed the data. YT and HZ conceived the study and wrote the paper. LC, HL, and SR performed the experiments. All of these authors approved the manuscript.

## Supporting information


**Fig. S1.** Sox4 and Sall4 are irrelevant to DANCR regulation in response to Ara‐C treatment.
**Fig. S2.** DANCR does not affect the expression of Beclin‐1, ATG5 and ATG7 in HL‐60 cells.
**Fig. S3.** ATG16L1 silencing and autophagy inhibitors recover DANCR‐conferred Ara‐C resistance.
