# Supplementary material for: Long noncoding RNA DANCR confers cytarabine resistance in acute myeloid leukemia by activating autophagy via the miR‐874‐3P/ATG16L1 axis
Source: Mol Oncol. 2021 Feb 27;15(4):1203–16. doi: 10.1002/1878-0261.12661 (PMC8024725; doi:10.1002/1878-0261.12661)
Supplement: Supplementary file 1 — Fig. S1. Sox4 and Sall4 are irrelevant to DANCR regulation in response to Ara‐C treatment. Fig. S2. DANCR does not affect the expression of Beclin‐1, ATG5 and ATG7 in HL‐60 cells. Fig. S3. ATG16L1 silencing and autophagy inhibitors recover DANCR‐conferred Ara‐C resistance. [file MOL2-15-1203-s001.pdf]

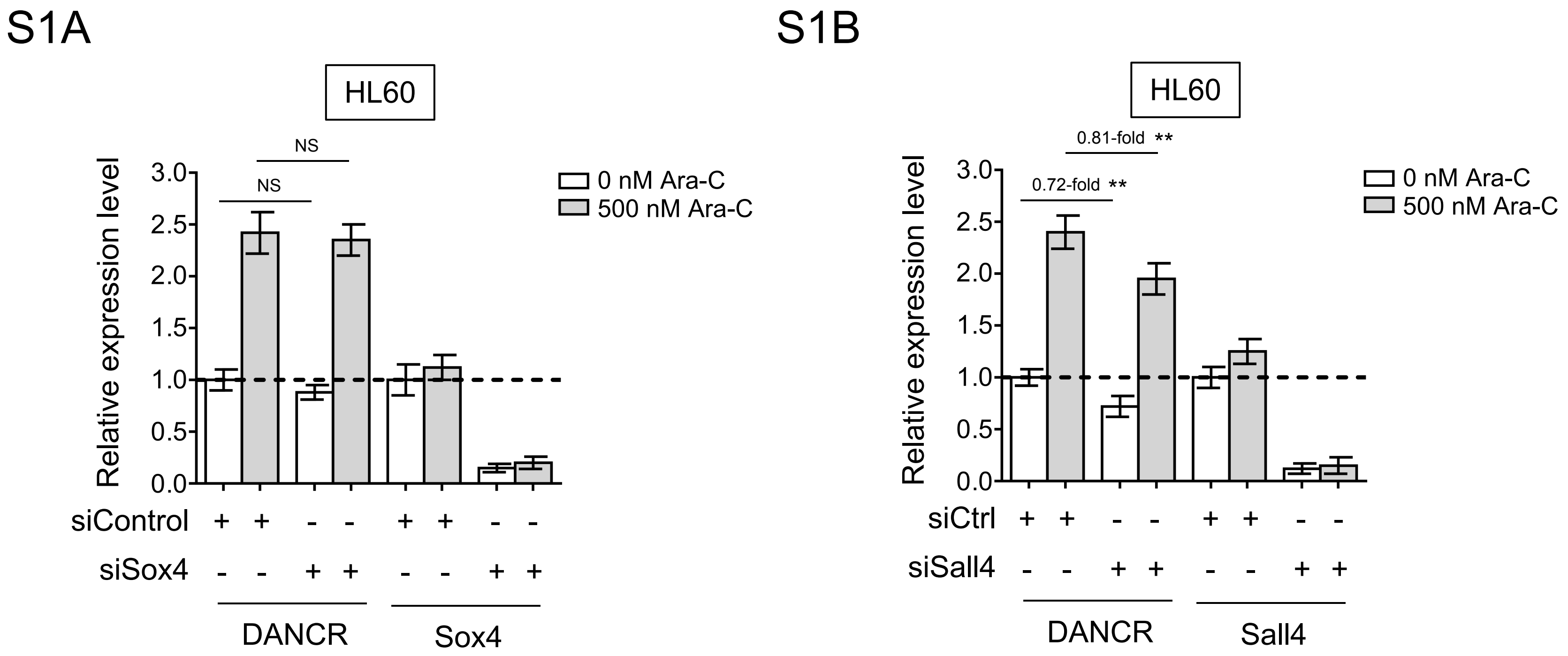

Supplementary Figure 1. Sox4 and Sall4 are irrelevant to DANCR regulation in response to Ara-C treatment

(A) Human AML HL60 cells were transfected with siCtrl or siSox4 (A) or siSall4 (B). Two days after transfection, cells were further treated with 0 or 500 nM Ara-C for 24 h. The expression of DANCR was determined by qRT-PCR analysis. β-actin was used as an endogenous control. The results are expressed as relative to vehicle treatment. Each column represents the value from 3 replicates. All data are mean ± SD. Data were analyzed using Student's t-test. **, P<0.01; NS, not significant.

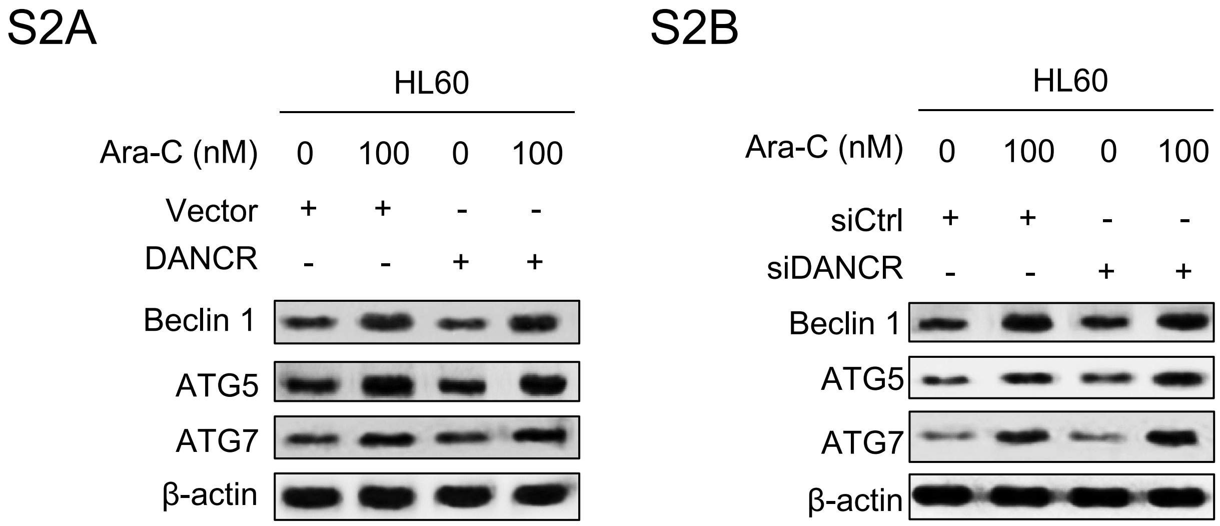

Supplementary Figure 2. DANCR does not affect the expression of Beclin-1, ATG5 and ATG7 in HL-60 cells
(**A**) HL60 cells were transfected with empty vector pcDNA3.1 or pcDNA3.1 expressing DANCR for 48 h, and then treated with or without 100 nM Ara-C for 24 h. The expression of Beclin-1, ATG5 and ATG7 was measured by immunoblotting. (**B**) HL60 cells were transfected siRNA targeting scrambled sequence (siCtrl) or DANCR (siDANCR #2) for 48 h, and then treated with or without 100 nM Ara-C for 24 h. The expression of Beclin-1, ATG5 and ATG7 was measured by immunoblotting. The images are representative from 3 independent assays.

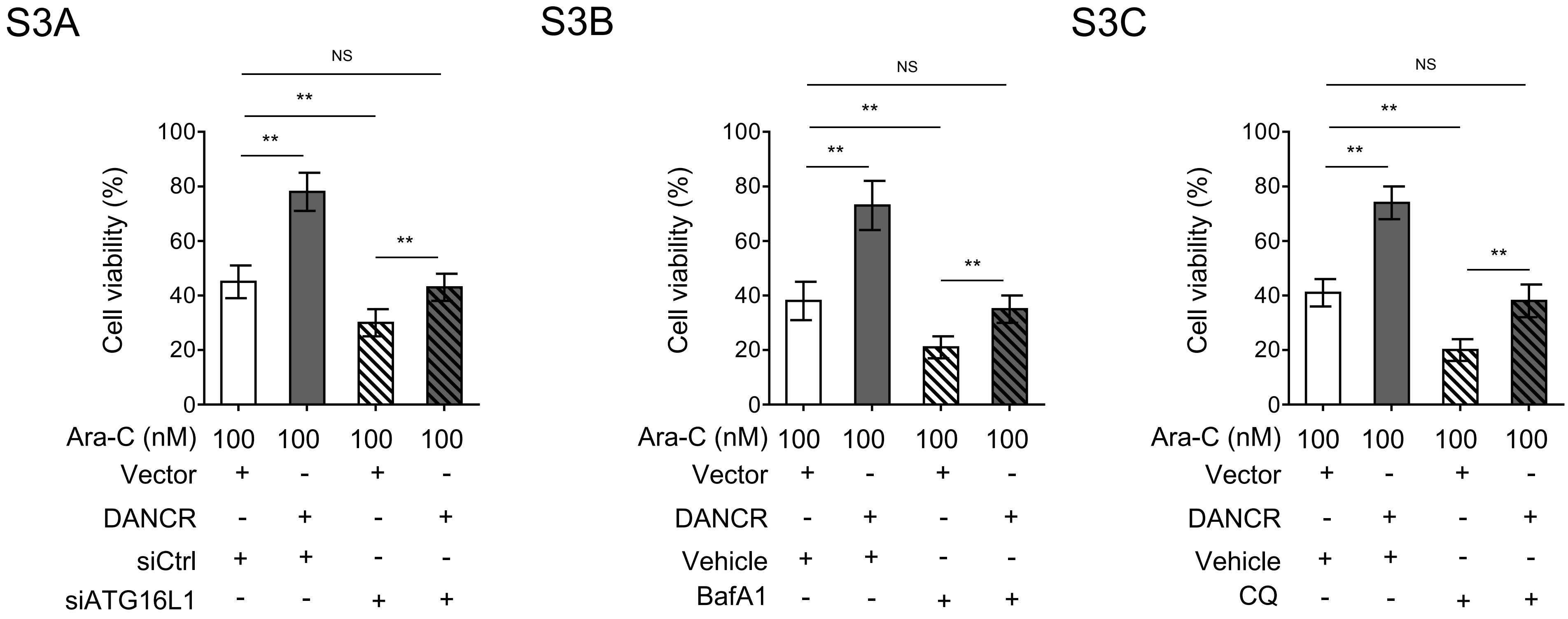

Supplementary Figure 3. ATG16L1 silencing and autophagy inhibitors recover DANCR-conferred Ara-C resistance
(A) HL60 cells were co-transfected empty vector pcDNA3.1 or pcDNA3.1 expressing DACR with siRNA targeting scrambled sequence (siCtrl) or ATG16L1 (siATG16L1) for 48 h in the presence or absence of 100 nM Ara-C. Cell viability was analyzed by MTT assay. The results are expressed as percentage of vector group (%). (B-C) HL60 cells were treated with 200 nM BafA1 (B) or 10 μM CQ (C) in the presence or absence of 100 nM Ara-C for 48 h. Cell viability was analyzed as in (A). BafA1, Bafillomycin A1; CQ, chloroquine. Data are mean ± SD and compared using Student's t-test. n = 3. **, P<0.01; NS, not significant.
